# Mycotic Keratitis in a Tertiary Hospital in Northeastern Malaysia

**DOI:** 10.4274/tjo.galenos.2020.57609

**Published:** 2020-12-29

**Authors:** Siva Chitamparam, Thiam-Hou Lim, Evelyn Tai, Mohtar Ibrahim

**Affiliations:** 1 Universiti Sains Malaysia School of Medical Sciences, Health Campus, Department of Ophthalmology, Kelantan, Malaysia

**Keywords:** Keratitis, fungi, Fusarium

## Abstract

**Objectives:**

To identify the clinical profile, etiology, and outcome of culture-positive mycotic keratitis in a tertiary referral centre in the northeastern part of Malaysia.

**Materials and Methods:**

A retrospective review of all patients with culture-positive mycotic keratitis in Hospital Universiti Sains Malaysia over a 3-year period, from January 2015 to December 2017.

**Results:**

This study included 27 eyes of 27 patients treated for mycotic keratitis based on a positive fungal culture. The most common predisposing factor was ocular trauma, in 22 patients (81.5%). Eleven patients (40.7%) had a presenting visual acuity worse than 6/60, due to central ulcer involvement. Approximately half of these (6 patients) experienced visual improvement post-treatment. *Fusarium* spp. was the most common fungus isolated (37%), followed by non-sporulating fungi and *Curvularia* spp. Three patients (7.4%) had corneal microperforations, which healed after gluing and bandage contact lens application. One patient (3.7%) required tectonic penetrating keratoplasty and 1 patient (3.7%) underwent evisceration. The final visual acuity was 6/18 or better in approximately half (14 patients) of our cohort and worse than 3/60 in approximately 20% (5 patients).

**Conclusion:**

Mycotic keratitis occurred mainly in males and secondary to ocular trauma. The most common organism isolated was *Fusarium* spp. Although treatment may improve vision, the visual outcome is guarded.

## Introduction

The cornea is the transparent, protective outer layer of the eye and the main structure responsible for focusing light rays onto the retina. Microbial keratitis refers to corneal inflammation secondary to infectious causes. The causative organisms of microbial keratitis include bacteria, viruses, and fungi. Fungal keratitis, also known as mycotic keratitis, is one of the leading causes of blindness, and remains the most challenging of all microbial keratitis.^1,2^ The incidence of fungal keratitis is significantly higher in developing countries, likely due to its close association with vegetative trauma in agricultural societies.^2,3^ Although reports on infectious keratitis are not uncommon, variations in causative organisms and their antimicrobial susceptibility among different study populations underscores the need for local data, which may provide individualized risk factor analysis and predict treatment outcomes.^1^ The Asia Cornea Society Infectious Keratitis Study (ACSIKS) included eight Asian countries (India, China, Japan, South Korea, Taiwan, Thailand, Philippines, and Singapore) and analyzed the risk factors, microbiology, and outcomes of infectious keratitis in Asian countries. Unfortunately, Malaysia was not included in ACSIKS. Our study thus aimed to identify the clinical profile, etiology, and outcomes of culture-positive mycotic keratitis in a tertiary referral center in the northeastern part of Malaysia.

## Materials and Methods

This was a retrospective review of all culture-positive fungal keratitis in Hospital Universiti Sains Malaysia over a 36-month period, from January 2015 to December 2017. Exemption from ethical review was obtained from the Human Research Ethics Committee of Universiti Sains Malaysia, as it was a fully anonymized study. The study adhered to the tenets of the Declaration of Helsinki.

All culture-positive fungal keratitis patients were identified from the database of corneal ulcers in the Department of Ophthalmology, Hospital Universiti Sains Malaysia. This database included both patients seen in the ophthalmology outpatient clinic and those who required inpatient care. All patients were examined under slit lamp and corneal scrapings were obtained under aseptic technique by using a sterile 21-gauge needle after the instillation of a local anesthetic (proparacaine hydrochloride 0.5%). The culture plates we used were blood agar, chocolate agar, MacConkey agar, and Sabouraud dextrose agar. The culture plates were then sent to our microbiological laboratory for incubation. Each patient’s treatment regimen was individualized based on the clinical features and progression of the ulcer.

Data obtained from the hospital medical records included demographic features, clinical comorbidities, precipitating factors, location of ulcer, presenting and final visual acuity, organism cultured, and treatment. Presenting and final visual acuity was measured with a Snellen chart placed at 6 meters, with spectacle correction in the presence of refractive errors. Presenting visual acuity was defined as the documented visual acuity during first consultation, while final visual acuity was defined as the visual acuity at least 6 months after ulcer healing. Patients with incomplete data were excluded from the study.

## Results

A total of 136 patients were diagnosed with infective keratitis during this period. Among these, 27 eyes of 27 patients were diagnosed as fungal keratitis based on a positive fungal culture. Their median age was 54 years (range: 21-77 years). Approximately 80% were male. The most common predisposing factor for developing fungal keratitis was trauma (81.5%). Other demographic features are shown in Table 1.

Table 2 shows the presenting visual acuity of our cohort. Ten patients (37.0%) had a presenting visual acuity of 6/18 or better, while 11 patients (40.7%) had a presenting visual acuity worse than 6/60. All patients in the latter group had centrally-located ulcers (Table 3). Among them, 6 (54.5%) experienced visual improvement after treatment; 1 (16.7%) achieved a final visual acuity of 6/18 or better, while 5 (45.5%) had a final visual acuity between 6/18 and 6/60.

*Fusarium* was the most commonly isolated genus (n=10, 37%), followed by non-sporulating fungi (n=5, 18.5%) and *Curvularia* (n=5, 18.5%). Most of our cohort were treated with dual topical antifungals (topical amphotericin B and topical fluconazole), as shown in Table 4. Choice of treatment was based on the clinical appearance and progression, as well as response to treatment. The ulcers in approximately one-fifth of cases healed with antibiotic and antiviral therapy only; antifungals were not started as the initial clinical appearance was not suggestive of fungal infection. In these patients, we noted that the ulcers were peripheral, and the presenting vision correspondingly good. One patient whose corneal scraping initially grew *Staphylococcus aureus* required evisceration; culture of the eviscerated tissue later revealed *Candida* sp.

Out of the 5 cases with perforation, three were caused by *Fusarium* spp. and 2 by *Candida*. However, both *Candida* cases resulted in a vision level of no light perception, while the one patient who needed evisceration was the patient with mixed infection with *S. aureus*.

Duration of treatment was not analyzed, as we believed that confounding factors such as questionable compliance to treatment post-discharge rendered it irrelevant. However, we observed that most of the patients (n=16, 59.3%) required a long duration of hospitalization (more than 14 days) for the initial treatment (minimum 3 days, maximum 44 days, median 15 days).

## Discussion

Fungal keratitis is a global public health problem which is especially challenging for ophthalmologists in developing counties.^1,2^ It is a particular burden in tropical countries, where it may comprise up to 67% of infectious keratitis.^3^ Obstacles to successful management include delayed diagnosis, longer healing times, a higher risk of corneal perforation, and overall worse visual outcome.^2,3^ Our case series presents the clinical profile and etiology of mycotic keratitis in a tertiary referral center in northeastern Malaysia. We also evaluated the treatment regimens, sequelae, and visual outcomes of mycotic keratitis in this cohort.

The median age in our cohort was 54 years. This is similar to the age distribution reported in developed countries.^3,4^ In developing countries, however, teenagers and young adults appear to be at greater risk, possibly due to occupational factors.^5^ Males were predominant, which is in accordance with the literature.^6,7^ Most of our patients were farmers; being a farmer, laborer, or unemployed has been shown to be associated with increased risk of fungal keratitis.^5^ These findings may also explain why patients in rural areas are at higher risk of fungal keratitis than those in urban areas.^5^

**Figure 1:**
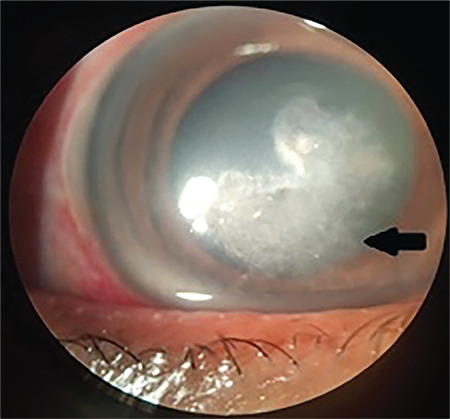
Anterior segment photo showing a typical fungal infiltrate with feathery edges (arrow)

**Figure 2:**
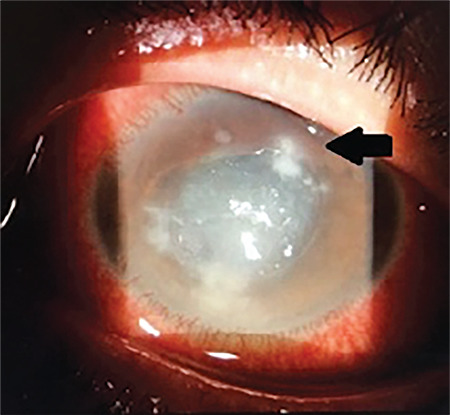
Anterior segment photo demonstrating satellite lesions (arrow)

Our study showed that trauma was the most common precipitating factor for mycotic keratitis. Immunocompromise and trauma, particularly vegetative, are the most common factors reported in association with fungal keratitis.^5,8,9^ Presence of risk factors appears to be common with fungal keratitis, as in our series.^7^ Diabetes mellitus has been shown not only to be a risk factor, but also to affect the severity of fungal keratitis.^10^ Typical clinical signs of fungal ulcers such as feathery infiltrate (Figure 1), satellite lesions (Figure 2), endothelial plaque, and ring infiltrate are not present in all cases during the initial stages. Factors affecting the timing of onset of mycotic keratitis after trauma include the type of organism, the size of epithelial defect, and host immune system.^7^ Dalmon et al.^11^ reported that corneal specialists were able to correctly differentiate bacterial from fungal etiology by visual inspection in only 66% of cases. Thus, in the presence of risk factors, clinical suspicion is crucial for timely management of fungal keratitis.

The etiology of fungal keratitis shows geographical variations. We found *Fusarium* sp. to be the most common organism isolated, followed by *Curvularia* spp. and non-sporulating fungi (mycelia sterilia). The two most common fungi causing fungal keratitis worldwide appear to be *Aspergillus* spp. and *Fusarium* spp.^1,8,12,13,14,15^, while *Curvularia* spp. have been cultured in Australia and the United States of America.^15,16^

Intrastromal amphotericin B was injected in a few of our patients with severe fungal keratitis. Hu et al.^17^ observed that a combination of intrastromal and intracameral amphotericin B is safe and effective for refractory fungal keratitis. However, a randomized controlled trial of intracameral amphotericin B in fungal keratitis found no benefit of this regimen over topical therapy.^18^ A Cochrane review on medical interventions for fungal keratitis evaluated various treatment regimens including voriconazole, itraconazole, and natamycin, concluding that natamycin is more effective than voriconazole in the treatment of fungal ulcers.^19^ Unfortunately, natamycin is unavailable in Malaysia, while voriconazole is prohibitively expensive. This is the reason that most of our patients were on combined topical amphotericin B and topical fluconazole, with oral fluconazole added in severe cases. We do not use corticosteroids in the management of fungal keratitis, as we are of the opinion that corticosteroids increase fungal replication by lowering host resistance.^10^ This prolongs the fungal clearance period, thus delaying the clinical response.^17^

We observed that approximately 60% (16 out of 27) of our cohort improved, with a third of our patients achieving a visual acuity of 6/12 or better. Those with better initial presenting vision had better final visual acuity, which is in keeping with the literature.^8^ Prajna et al.^20^ reported that large infiltrate size and severe fungal ulcers with presence of hypopyon were significantly associated with higher risk of corneal perforation. Other factors like visual acuity, epithelial defect size, baseline culture positivity, type of organism, and duration of symptoms are not strong predictors of corneal perforation.^20^ In our one patient who required evisceration secondary to corneal perforation, the progression of her disease was attributed to a missed diagnosis of fungal keratitis, as her initial culture grew *S. aureus, *while the histopathology of the eviscerated specimen grew *Candida* sp. This is a reminder that one should consider mixed infection in cases of poor response to treatment despite a positive culture and sensitivity to prescribed antibiotics.

Our study provides a comprehensive overview of the clinical profile, etiology, and outcome of culture-positive mycotic keratitis in a tropical center. Strengths of our study over other published studies (Table 5) are its documentation of visual acuity and evaluation of the relationship between ulcer location and final visual acuity. Additionally, we show that small peripheral ulcers may recover without antifungal therapy, as occurred in 20% of our patients. Spontaneous resolution of small fungal keratitis has been attributed to host immune response and inhibition of fungal growth by use of topical fluoroquinolones.^21^

### Study Limitations

There are several limitations of our study. First, as our sample was restricted to those with a positive fungal culture, our conclusions may not apply to fungal keratitis identified by other methods, such as *in vivo* corneal confocal microscopy. Secondly, due to its retrospective nature, there was lack of a standardized treatment protocol for mycotic keratitis; thus, we are unable to make any inferences regarding the comparative efficacy of different treatment approaches. Future research should involve a prospective, multicenter study to determine the optimal management of mycotic keratitis.

## Conclusion

The most common organism causing mycotic keratitis in our cohort was *Fusarium*. Ocular trauma was the main predisposing factor. Peripheral ulcers may resolve without antifungal therapy, while central ulcer involvement has a worse visual prognosis. Dual topical antifungal agents were the main treatment initiated. The visual outcome generally improved post-treatment. A strong clinical suspicion of fungal or mixed infection is important in cases of poor treatment response, as a missed diagnosis of mycotic keratitis can have severe visual consequences.

## Figures and Tables

**Table 1:**
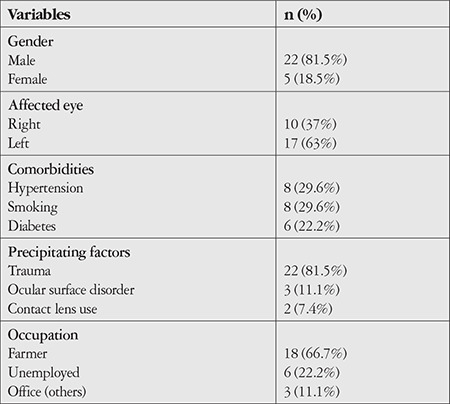
Demographics of the study sample

**Table 2:**
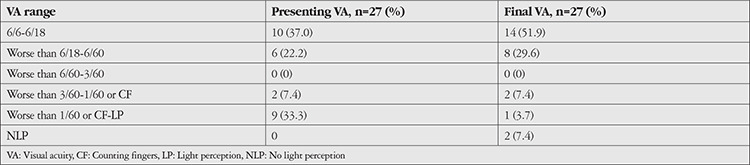
Presenting and final visual acuity of study subjects

**Table 3:**
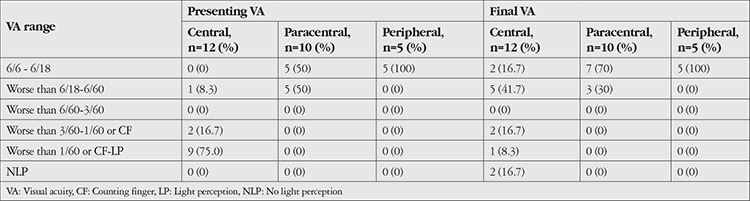
Presenting and final visual acuity based on ulcer location

**Table 4:**
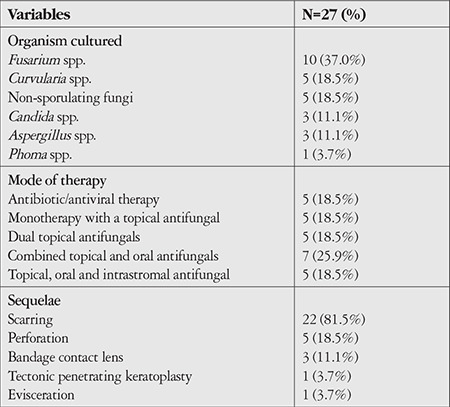
Organism, mode of therapy, and sequelae

**Table 5:**
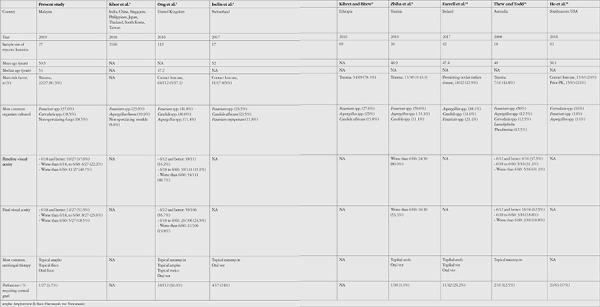
Comparison of clinical profiles, etiologies, and outcomes of mycotic keratitis in published studies
